# Taxing sugar sweetened beverages in Indonesia: Projections of demand change and fiscal revenue

**DOI:** 10.1371/journal.pone.0293913

**Published:** 2023-12-29

**Authors:** Agus Widarjono, Rifai Afin, Gita Kusnadi, Muhammad Zulfiqar Firdaus, Olivia Herlinda

**Affiliations:** Center for Indonesia’s Strategic Development Initiatives (CISDI), Jakarta, Indonesia; University of Sydney, AUSTRALIA

## Abstract

The global trend of diets high in sugar sweetened beverages (SSB) is associated with a high risk of obesity and non-communicable diseases (NCDs). To reduce SSB consumption on a population level, SSB taxes have become a popular policy solution. In Indonesia, although the prevalence of obesity has doubled in the past decade (11.7% in 2010 to 21.8% in 2018), SSB taxes have not yet been implemented. Utilizing the 2021 Indonesian household socioeconomic survey (SUSENAS), this study estimated price elasticities and projected the plausible effects of implementing an SSB tax on consumers’ demand for SSBs and the associated government revenue using the Quadratic Almost Ideal Demand System (QUAIDS) model. Five SSB groups were studied: 1) manufactured liquid milk; 2) sweetened condense milk; 3) instant coffee; 4) tea drinks and fizzy drinks with CO_2_; 5) fruit juices, “health” drinks, and energy drinks. The overall results showed that the non-milk SSB groups were price elastic. Probing deeper into the substitutions for SSB across categories, we found both substitutionary and complementary effects. Our analysis revealed that increasing SSB prices by 20% would reduce the demand for SSBs on average by 17.5% (14.3%-18.6% for each SSB group) and generate additional state revenue up to IDR 3,628.3 billion per year (approximately US$ 238.5 million or 0.2% of total tax revenue in 2022). Considering the health and economic impacts of high consumption of SSBs, this study provides empirical evidence that imposing taxes on SSBs could be an effective measure to reduce public consumption and to generate tax revenue for financing health programs that address obesity and NCDs in Indonesia.

## Introduction

The epidemic of obesity has been of global concern as its prevalence has nearly tripled in the last four decades [1]. In Indonesia, 21.8% of the total population is obese, double the previous decade [2, 3].

Consumption of sugar sweetened beverages (SSB) has been associated with obesity and a higher risk of non-communicable diseases (NCDs) [4, 5] including diabetes, cardiovascular diseases, and cancers, as well as premature mortality [6–8]. Data from Indonesia’s National Economic Survey reveals that consumption of SSBs in Indonesia has increased 15 times in the last two decades [9]. A survey conducted in 2016 also reported that 62% children, 72% adolescents, and 61% adults in Indonesia consumed SSBs at least once a week [10].

A systematic review conducted by the World Health Organization (WHO) showed that taxing sugary drinks is effective in reducing the consumption of SSBs [11]. As of today, more than 50 countries globally, including low and middle-income countries (LMICs) have implemented this policy [12]. In Indonesia, the discussion of SSB taxes was initiated in 2016 by the Ministry of Finance, but as of February 2023, the policy remains unimplemented [13].

The potential impacts of the increase in price (through SSB taxation) on consumption have been evaluated through estimations of elasticity. The estimates conducted in Mexico [14, 15], Colombia [16], and Thailand [17] suggest that SSBs are elastic, and therefore, raising the price of SSBs reduces consumer demand for SSBs, and leads to reduced consumption. Based on our review of the literature, price elasticity estimate analyses for SSBs have not yet been conducted in Indonesia. This study seeks to understand the price elasticity of SSBs in Indonesia and devise elasticity estimates to understand the impact of increasing SSB prices on consumer demand. This study also estimates the potential increase of annual government revenue generated from SSB excise tax.

## Methods

### Elasticities estimation

We used the Indonesia National Household Socioeconomic Survey (SUSENAS) 2021 data, which recorded a total consumption of 188 different types of food and beverage expenditures of 340,032 households across 34 provinces over the course of one week in the year [18]. We included five categories of beverages in our study: (1) manufactured liquid milk; (2) sweetened condense milk; (3) instant coffee (sachets); (4) tea drinks and fizzy drinks with CO_2_; and (5) fruit juices, “health” drinks, and energy drinks. We assumed these beverages are categorized as SSBs since they typically contain sugar, either added or naturally occurring [19]. While sweetened condensed milk is usually not considered SSB since it is typically used for other purposes (usually being added to or mixed with other foods and beverages), we added it to our study since Indonesia’s government also proposed sweetened condensed milk in its SSB tax proposal [20]. We also included bottled mineral water as a potential substitute for SSBs since a large portion of Indonesians rely on it for hydration due to a lack of access to safe drinking water [21]. Bottled mineral water is also very accessible and can be a healthier alternative for hydration at any time since it contains zero sugar.

To estimate the price and cross price elasticity of SSBs, we used the Quadratic Almost Ideal Demand System (QUAIDS) [22] as follows:

$$w_{i}=\alpha_{i}+\sum_{j=I}^{n}\gamma_{ij}\ln p_{j}+\beta_{i}\ln(\frac{X}{a(P)})+\frac{\lambda_{i}}{b(P)}{\left(\ln[\frac{X}{a(P)}]\right)}^{2}\forall i\in I$$ (1)

Where *i* and *j* are the types of drink, *w_i_* is the budget share allocated to buy drink *i*, *p_j_* is the price of drink *j*, X is the total household expenditure for the drink, *I* is the set of all beverage groups, *b(P)* is the price aggregator given by:

$$b(p)=\underset{j\in I}{\prod }p_{j}^{\beta_{j}}$$ (2)

and *a*(*P*) is the price index calculated by:

$$\ln a(p)=\alpha_{0i}+\sum_{j\in k}\alpha_{j}\ln p_{j}+\frac{1}{2}\sum_{l\in 1}\sum_{j\epsilon 1}\gamma_{lj}\ln p_{l}\ln p_{j}$$ (3)

We controlled for demographic characteristics by adding demographic variables in the intercept of Eq (1), including household size and head of household characteristics (age, gender, marital status, occupation, and education level).

Since SUSENAS data do not present the price of any beverage products, we calculated the price by regressing a unit value with household income and a regional dummy as performed by Jensen & Manrique [23]. The unit value is obtained by dividing the consumption value (IDR) by the quantity consumed. For household income, we used the total household expenditure as a proxy.

By solving the Eq (1), the price elasticity is therefore expressed as:

$$e_{ij}=\frac{1}{W_{i}}\{\gamma_{ij}-(\beta_{i}+\frac{2_{i}}{b(P)}[\ln(\frac{X}{a(P)})])(\alpha_{ih}+\sum_{j=1}^{n}\gamma_{ij}\ln p_{j})-\frac{\beta_{ij}}{b(P)}{\left(\ln[\frac{X}{a(P)}]\right)}^{2}\}-d_{ij}$$ (4)

Furthermore, as SUSENAS only captures the household consumption of SSBs over one week, we inferred the elasticity using a censoring approach introduced by Hein & Wessells [24] which mimics Heckman’s two step approach [25] to accurately estimate variation in purchases of SSBs throughout the year.

### SSB demand projection

The demand projection was done by estimating the demand changes based on the computed price elasticities of the four SSB categories. Any SSB demand is likely affected by changes of the price, own-price elasticity, and/or cross-price elasticity. The price increase impact on SSB demand is therefore calculated as:

$$\Delta SSB_{j}=\%\Delta \;price*own\;price\;elasticity+\%\Delta \;price*cross\;price\;elasticity\;$$ (5)

We estimated the demand projection based on the 20% price increase, the rate shown to effectively reduce SSB consumption based on systematic reviews [26, 27]. In addition, 20% tax rate is also set as a general SSB tax recommendation by the WHO and is commonly used in other SSB price elasticity studies conducted in other countries [11, 15–17].

### Government revenue projection

In projecting the government revenue, we integrated the SUSENAS data with the forecasted sales data from Global Data [28] for the following categories of SSBs: 1) manufactured liquid milk, 2) sweetened condense milk, 3) tea drinks, 4) fizzy drinks with CO_2_, 5) fruit juices, 6) “health” drinks, and 7) energy drinks. Since SUSENAS data merged several beverages into one group instead of individually (e.g., fruit juice, “health” drinks, and energy drinks), we assumed that the price elasticity of individual beverages are similar to that of the category. In addition, instant coffee was excluded from this analysis because the forecasted sales data were not available.

We used two different tax designs (ad valorem and specific volumetric tax) in projecting the tax revenue: 1) 20% ad valorem [26, 27], and 2) three scenarios of specific volumetric tax proposed by the Indonesian Ministry of Finance (MoF) in 2021 [20] (see Table 1).

**Table 1:** Tax Scenarios proposed by the MoF in 2021 for SSBs and concentrated SSBs.

Sugar content	Lump sum tax (IDR per liter)		
	Scenario I	Scenario II	Scenario III
<5 gr/240 ml	0	0	0
5–20 gr/240 ml	1,500/L	2,000/L	4,200/L
>20 gr/240 ml	2,500/L	2,771/L	4,200/L

Source: Indonesian Ministry of Finance’s socialization on *Undang-Undang Harmonisasi Peraturan Perpajakan (Klaster Cukai) & Rencana Kebijakan Ekstensifikasi Cukai*, dated 17 December 2021

The tax revenue of the 20% ad valorem tax (Eq 6) is calculated as the forecasted volume of sales multiplied by the ad valorem tax rate (20%) using an average price per liter across all SSB categories and assuming complete pass through of the tax to the consumer.


$$\mathrm{Tax}\;{\mathrm{revenue}}_{i}=\mathrm{Forecasted}\;\mathrm{Sales}*\mathrm{ad}\;\mathrm{valorem}\;\mathrm{tax}\;\mathrm{rate}*\mathrm{average}\;\mathrm{price}\;\mathrm{of}\;\mathrm{SSB}$$ (6)

The volumetric tax revenue is calculated as:

$$Tax\;revenue_{i}=Forecasted\;Sales*lump\;sum\;tax\;rate\;$$ (7)

## Results

### Descriptive statistics

Table 2 provides the descriptive statistics of the household consumption of SSB and mineral water based on the SUSENAS data. Among the 340,032 households surveyed, the most commonly consumed SSB was instant coffee (29.4%) and the least consumed SSB was manufactured liquid milk (5.7%). Mineral water, the only unsweetened beverage in this study, was consumed by 20.5% of the households that spent, on average, IDR 44,129 (US$ 2.9) per household per month on that beverage. While manufactured milk was the least consumed beverage, it was the one households spent the most money on (on average IDR 84,285 or US$ 5.5 per month). In contrast, households spent the least (on average IDR 35,999 or US$ 2.4) on tea drinks and fizzy drinks with CO_2_. In terms of the household’s average serving per month, instant coffee was the most consumed (29 servings) and sweetened condense milk was the least (5 servings). Table 2 also shows that the most expensive beverage was sweetened condense milk, and the cheapest beverage was instant coffee.

**Table 2:** Descriptive statistics on household consumption of SSB and mineral water.

Beverages	Proportion of Households Consuming (%)	Average Household Monthly Expenditure (IDR)	Average Household Monthly Consumption (Serving)	Average Price Per Serving (IDR)
Manufactured liquid milk (± 250 ml)	5.7	84,285	19	4,436
Sweetened condensed milk (397 gr)	22.2	51,119	5	10,224
Instant coffee (20 gr)	29.4	40,577	29	1,399
Tea drinks, fizzy drinks with CO_2_ (± 250 ml)	19.6	35,999	12	2,999
Fruit juices, “health” drinks, energy drinks (± 200 ml)	17.9	42,370	13	3,259
Bottled mineral water (± 200 ml)	20.5	44,129	9	4,903

Source: SUSENAS 2021

### Elasticities of SSB

#### Own-price elasticity

Own-price elasticity shows the sensitivity of price changes on expenditures, in which the absolute value of one implies that expenditure and price changes vary in the same proportions. Elastic demand (n>1) means the decreasing expenditure of a product is bigger than the price increase. In other words, the demand of the product is sensitive to price changes. In contrast, inelastic demand (n<1) means the decreasing expenditure of a product is less than the price increase, indicating the demand of the product is less sensitive to price changes. The elasticity also infers whether or not the product is perceived to be essential by the consumer. Inelastic products are necessary goods and non-substitutable, while elastic products are easily substituted with other goods which are cheaper or fulfill a similar need.

Table 3. Panel (a) shows the own-price elasticity of each SSB category. Overall, the demand for manufactured liquid milk and sweetened condensed milk are price inelastic (-0.69 and -0.33), meaning that when the price increases, the decrease in expenditure of manufactured liquid milk and sweetened condensed milk will be smaller than the increased price. In other words, people will consume them but not at the same quantity as they would if they were at a lower price. In contrast, the demand for other categories of SSBs are price elastic (-1.38 for instant coffee, -1.18 for tea drinks and fizzy drinks with CO_2_, and -1.11 for fruit juices, “health” drinks, and energy drinks), because these products are not necessities and are easily substitutable when and if their prices increase.

**Table 3:** Elasticities of SSBs.

	**Own-price elasticity**									
Beverages	Household income levels				Regions		Age of head of household		Years of schooling of head of household	
	All	25%	50%	75%	Urban	Rural	≤50	>50	≤12	>12
Manufactured liquid milk	-0.69	-0.77	-0.75	-0.73	-0.60	-0.74	-0.66	-0.73	-0.70	-0.12
Sweetened condensed milk	-0.33	-0.38	-0.36	-0.35	-0.35	-0.32	-0.33	-0.35	-0.33	-0.33
Instant coffee	-1.38	-1.36	-1.37	-1.38	-1.37	-1.39	-1.39	-1.37	-1.38	-1.41
Tea drinks, fizzy drinks with CO_2_	-1.18	-1.17	-1.17	-1.17	-1.18	-1.17	-1.17	-1.18	-1.17	-1.19
Fruit juices, “health” drinks, energy drinks	-1.11	-1.11	-1.11	-1.11	-1.11	-1.11	-1.11	-1.11	-1.11	-1.11
Bottled mineral water	-0.62	-0.66	-0.65	-0.64	-0.59	-0.64	-0.63	-0.61	-0.62	-0.52

	**Cross-price elasticity**					
Beverages	Manufactured liquid milk	Sweetened condensed milk	Instant coffee	Tea drinks, fizzy drinks with CO_2_	Fruit juices, “health” drink, energy drink	Mineral water
Manufactured liquid milk	-	0.77	-0.57	-0.29	-0.39	0.46
Sweetened condensed milk	0.10	-	-0.63	-0.26	-0.19	0.42
Instant coffee	0.06	0.46	-	-0.17	-0.15	0.26
Tea drink, fizzy drinks with CO2	0.07	0.43	-0.37	-	-0.12	0.25
Fruit juices, “health” drinks, energy drinks	0.10	0.37	-0.40	-0.15	-	0.26
Bottled mineral water	0.11	0.76	-0.60	-0.27	-0.22	-

Source: Authors’ calculation

Furthermore, based on the availability of information in SUSENAS, we analyzed elasticities across different household characteristics. More elastic demand was observed for manufactured liquid milk and sweetened condensed milk among lower income households and instant coffee among higher-income households. In other words, lower income households put lower value on manufactured liquid milk and condensed milk; and higher value on instant coffee when their prices increase. Meanwhile, the elasticity of tea drinks, fizzy drinks with CO_2_, fruit juices, “health” drinks, and energy drinks are similar across different households’ income levels. This means lower and higher income households put the same value on these beverages.

In terms of urban and rural households, more elastic demand was observed for sweetened condensed milk, as well as tea and fizzy drinks with CO_2_ among urban households, and for manufactured liquid milk and instant coffee among rural households. Meanwhile, the elasticity of fruit juices, “health” drinks, and energy drinks is similar across urban and rural households.

Elasticities also vary by the age and education level of the head of household. In this regard, older heads of households (50+ years) have more elastic demand for manufactured liquid milk, sweetened condensed milk, tea drinks, and fizzy drinks with CO_2_ compared to those with younger heads of households (<50 years). In contrast, younger heads of households have a slightly more elastic demand for instant coffee. In terms of the level of education, the demand for instant coffee, tea drinks, and fizzy drinks with CO_2_ is more elastic among more educated heads of household (12+ years of schooling) than those with lower education (<12 years of schooling). Conversely, the demand for manufactured liquid milk is more inelastic among higher educated heads of household as opposed to those with lower-educated heads of household.

#### Cross-price elasticity

Cross-price elasticity indicates the sensitivity of expenditure on a product to changes in the price of another product. When the elasticity values are positive (n>0), the price increase of another product would raise the expenditure for that product, indicative of substitutability of both products. On the other hand, when elasticity values are negative (n<0), the price increase of another product would reduce expenditure for that product, indicating that the two products are complementary.

Table 3. Panel (b) presents the cross-price elasticity which measures the change in expenditure of a particular SSB category in relation to the price change of another SSB category.

The results indicated that manufactured milk and sweetened condensed milk are substitutable with instant coffee; tea drink and fizzy drinks with CO2; fruit juices,”health”drinks, and energy drinks; and mineral water. Similarly, mineral water is also substitutable with manufactured milk; sweetened condensed milk; instant coffee; tea drink and fizzy drinks with CO2; and fruit juices,”health”drinks and energy drinks. In contrast, instant coffee; tea drinks, fizzy drinks with CO2; fruit juices,”health”drinks and energy drinks are complementary to each other as well as to manufactured milk, condensed milk, and mineral water.

While coffee and tea are generally viewed as substitutes for each other, we found that the category of SSB that includes tea is complementary to instant coffee. The reason for this is that the expenditure of our data is at the household level, i.e., one member of the household might consume tea while the other members might consume coffee. According to our data, 56.4% of households that consumed instant coffee also consumed tea at the same time.

Although sweetened condensed milk is usually consumed by being mixed with other beverages such as coffee, tea, soda, etc. (therefore the relationship should be complementary), we found that the condensed milk is a substitute for other drinks in our analysis. This contradictory result could be driven by the fact that the majority of households that consumed other types of beverages did not consume sweetened condensed milk in our data. For example, 74.2% of the households that consumed instant coffee and 71.6% of the households that consumed tea and fizzy drinks did not consume sweetened condensed milk.

### Demand change projections for SSBs

Table 4 presents the projected demand for SSBs when their prices are increased by 20%. Overall, the results showed that a 20% increase in price would reduce the demand on average, by 17.5%, ranging from 14.3–18.6% reduction for each SSB category analyzed. The largest decrease in demand is projected to occur in the category of fruit juices, “health” drinks, and energy drinks, while the smallest one is for manufactured liquid milk. Based on the levels of household income, the projection indicates that when the SSB price rises, the demand of lower income households would decrease more than that of higher-income households. Furthermore, the demand of rural households, households with older heads of household, and lower educated heads of household would decrease more than their counterparts.

**Table 4:** Estimated percent changes in SSB categories’ demand when prices are increased by 20%.

Beverages	Income levels				Regions		Age of head of household		Years of schooling of head of household	
	All	25%	50%	75%	Urban	Rural	< = 50	>50	≤12	>12
Manufactured liquid milk	-14.32	-15.78	-15.40	-15.04	-12.72	-15.24	-13.86	-15.04	-14.42	-4.36
Sweetened condensed milk	-17.88	-18.10	-18.04	-17.96	-17.82	-17.92	-17.84	-17.94	-17.86	-17.72
Instant coffee	-18.30	-18.50	-18.46	-18.38	-18.30	-18.36	-18.30	-18.36	-18.34	-18.12
Tea drinks, fizzy drinks with CO_2_	-18.30	-18.42	-18.40	-18.36	-18.20	-18.40	-18.32	-18.32	-18.28	-18.06
Fruit juice, “health” drinks, energy drinks	-18.64	-18.72	-18.72	-18.70	-18.56	-18.74	-18.64	-18.66	-18.64	-18.58
Average changes in demand	-17.50	-17.90	-17.80	-17.69	-17.12	-17.73	-17.39	-17.66	-17.51	-15.37

Source: Authors’ calculation

### Government revenue projection

The projected additional government revenue from implementing a 20% SSB tax with full pass through in Indonesia are summarized in Table 5. The 20% ad valorem tax could generate IDR 3,407.2 billion per year (approximately US$ 223.9 million or 0.2% of total tax revenue in 2022). Meanwhile, based on three scenarios proposed by the MoF, scenario III could generate even more revenue, IDR 3,628.3 billion (approximately US$ 238.5 million) per year.

**Table 5:** Projection of government revenue.

				Sales after tax (million liters)				Additional fiscal revenue (IDR billion)			
SSB category	Sugar content (gr per 240ml)	Average price per liter (IDR)	Projected sales (million liters)	20% Ad valorem tax	Volumetric tax as per MoF Proposal			20% Ad valorem tax	Volumetric tax as per MoF Proposal		
					Scenario I (%)	Scenario II (%)	Scenario III (%)		Scenario I	Scenario II	Scenario III
Manufactured liquid milk	20.16	19,048	166.87	143.84	151.8	150.1	142.7	547.97	379.39	415.97	570.75
				(-13.80)	(-9.06)	(-10.04)	(-14.49)				
Sweetened condensed milk	12.7	28,273	135.13	126.1	132.7	131.9	128.7	713.02	199.10	263.86	514.96
				(-6.68)	(-1.77)	(-2.36)	(-4.73)				
Tea drinks	20.16	1,896	478.1	365.75	377	366.1	316.4	1,016.48	942.58	1 014.40	1 265.58
				(-23.50)	(-21.14)	(-23.43)	(-33.82)				
Fizzy drinks with CO_2_	20.16	13,896	225.3	172.35	177.7	172.5	149.1	479.01	444.18	478.03	596.39
				(-23.50)	(-21.14)	(-23.43)	(-33.82)				
Fruit juices	26.4	18,760	48.91	38.06	41.68	40.9	37.34	142.79	104.20	113.32	149.35
				(-22.20)	(-14.79)	(-16.40)	(-23.67)				
“Health” drinks	26.4	18,760	72.96	56.76	62.17	61	55.69	212.97	155.41	169.02	222.76
				(-22.20)	(-14.79)	(-16.40)	(-23.67)				
Energy drinks	26.4	18,760	101.05	78.62	86.1	84.48	77.13	294.97	215.26	234.10	308.54
				(-22.20)	(-14.79)	(-16.40)	(-23.67)				
Total estimated revenue (IDR billion)								3407.21	2440.12	2 688.71	3 628.33

Source: Authors’ calculation

## Discussion

This is the first known study in Indonesia to use nationally-representative data to compare price elasticity across different beverage categories determining the demand for SSBs, and the potential of price increases (through an SSB excise tax) on consumer demand and government revenue generation.

In this study we have demonstrated that the demand for SSBs (instant coffee, tea drinks, fizzy drinks with CO_2_, fruit juices, “health” drinks, and energy drinks) across different household characteristics are price elastic. Meanwhile, the demand for manufactured liquid milk and sweetened condensed milk are inelastic, which indicates their limited availability of substitutes (note: sweetened condensed milk is often used for or mixed with a few specific types of foods or beverages in Indonesia and is rarely substituted with other sweeteners such as liquid sugar, syrups, etc.). By estimating changes in demand using the computed elasticities, we simulated whether the overall public consumption of SSBs can be expected to decline. The results showed that a 20% price increase for SSBs could reduce demand by 17.5% on average and, in turn, reduce the SSB consumption on a population level.

Our analysis of cross-price elasticity of SSB categories found that increasing the price of SSBs while keeping the price of mineral water the same could also promote healthier behaviors, such as substituting SSBs with mineral water. Therefore, we recommend taxing all SSBs to ensure that there is no substitution between SSB categories.

The potential government revenue generated from the MoF proposed SSB tax of approximately IDR 3,628.3 billion per year (around US$ 238.5 million or 0.2% of total tax revenue in 2022) would be a significant gain for the Indonesian economy. This figure falls between the estimated range of IDR2,700–6,250 billion per year as estimated by MoF [29]. However, a systematic review has shown that there would be expected variations when the tax policy is implemented, including the sensitization around the policy, which products are impacted by the tax, whether the industry reformulates their products, and among other considerations [30].

Our results are consistent with previous studies on SSB price elasticity conducted in other countries including Mexico [14, 15], Colombia [16], and Thailand [17], where SSBs were found to be price elastic, and therefore, increasing SSB price would significantly reduce consumption, although the SSB categories in this study were not comparable due to limited data availability. The potential increase in government revenue generated from SSB tax is also reported in a previous study conducted in Colombia [16].

This study is not without limitations. The SUSENAS data merge several SSBs into two categories (tea drinks, fizzy drinks with CO_2_; and fruit juices, “health” drinks, energy drinks), so it was difficult to determine which drinks within a category were more elastic than the others in the category. The two other categories, manufactured liquid milk and instant coffee, were available as single product categories, and we would recommend moving forward that SUSENAS provides data on individual product categories instead of collapsing different beverages in a multiple beverage category. Another limitation is the exclusion of macroeconomics variables, such as inflation, on the impact of a potential SSB tax. High inflation, especially when an SSB tax is implemented as a lump sum tax (as proposed by MoH), could render the tax insignificant relative to the total increased price.

## Conclusion

Given our results, we recommend the government implement the scenario III proposed by MoF and design a tax scheme that technically guarantees the transfer of the tax to at least 20% increase of SSBs final price. We also recommend the scheme be carried out to all SSB products (manufactured liquid milk; instant coffee; tea drinks and fizzy drinks with CO2; and fruit juices, “health” drinks, and energy drinks) in the future market.

Our study contributes to the wider literature on the evidence-based priority setting for improving Indonesian public health. Specifically, it is expected to pave the way for further study on SSB taxation in Indonesia, particularly the potential implications for public health and macroeconomics. Future research should look at the potential long-term implications of SSB tax on health outcomes, and on the impact of inflation of different tax designs.
